# *Natura Non Facit Saltus:* The Adaptive Significance of Arginine Vasopressin in Human Affect, Cognition, and Behavior

**DOI:** 10.3389/fnbeh.2022.814230

**Published:** 2022-05-02

**Authors:** Bernard Crespi, Tanya Procyshyn, Mika Mokkonen

**Affiliations:** ^1^Department of Biological Sciences, Simon Fraser University, Burnaby, BC, Canada; ^2^Autism Research Centre, Department of Psychiatry, University of Cambridge, Cambridge, United Kingdom; ^3^Department of Biology, Kwantlen Polytechnic University, Surrey, BC, Canada

**Keywords:** cooperation, sociality, conflict, autism, loneliness, arginine vasopressin

## Abstract

Hormones coordinate internal bodily systems with cognition, affect, and behavior, and thereby influence aspects of social interactions including cooperation, competition, isolation, and loneliness. The adaptive significance and contextuality of oxytocin (OXT) and testosterone (T) have been well-studied, but a unified theory and evolutionary framework for understanding the adaptive functions of arginine vasopressin (AVP) remain undeveloped. We propose and evaluate the hypothesis that AVP mediates adaptive variation in the presence and strength of social and sociosexual salience, attention and behavior specifically in situations that involve combinations of cooperation with conflict or competition. This hypothesis can help to explain the ancestral, original functions of AVP-like peptides, and their continuity with the current roles of AVP, for humans, in male-male competition, male-male reciprocity, male-to-female pair bonding, female-female interactions, social integration, and social attention and anxiety. In this context, social isolation and loneliness may be mediated by reduced abilities or interests in navigation of social opportunities and situations, due in part to low AVP levels or reactivity, and in part to reductions in levels of OXT-mediated social reward.

## Introduction

Hormones coordinate physiological states with cognition and emotion, and thereby orchestrate the complex adaptive contextuality of human behavior. Human behavior is thus optimized, in each situation, toward maximizing one’s inclusive fitness, within the challenges and constraints imposed by other individuals who are trying to do the same for themselves (Alexander, 1987). Human social interaction often comprises mixtures of cooperation with conflict, whose expression is determined by variables including kinship, leverage, physical power asymmetries, aspects of personality, and information (Lewis, 2002; Watts, 2010; Summers and Crespi, 2013). In turn, the nature of social interactions (or lack thereof), and one’s abilities to increase inclusive fitness, drive the development and expression of mental health conditions, aversive mental states including loneliness, and other departures from mental wellbeing (Nesse, 2019).

Two peptide hormones, oxytocin (OXT) and arginine vasopressin (AVP), are explicitly social in their cognitive, emotional and behavioral effects (McCall and Singer, 2012; Mitra, 2021). The evolved, adaptive functions of OXT are generally well-understood: this hormone controls feeling and expression of warmth, love, and cooperative social bonding in dyads and groups, supports emotional empathy, kinship, and altruism, and reduces stress (Crespi, 2016). These functions derive, ultimately and evolutionarily, from OXT’s ancestral roles among early mammals in the smooth muscle contraction necessary for successful parturition, and lactation, and in supporting the neurological basis of mother-child bonding. The predominantly cooperative and positive nature of OXT within pairs or small groups (aside from maternal defense of young), was, over time, co-opted to encompass a broader range of social interactions, including sociosexual bonding between mates, father-child interactions, and bonding within large social groups united by extended kinship networks and shared culture (Crespi, 2016).

In contrast to OXT, a unified conceptual framework for the evolved, adaptive functions of AVP remains almost entirely undeveloped. Instead, myriad studies that have applied endocrinological, neurological, and behavioral methods have cataloged a diverse range of specific functions for AVP’s behavioral effects, including stress, social attention, social recognition, social memory, reciprocity, male to female pair bonding, agonism among males, affiliation among females, and play among juveniles, among others (Young and Wang, 2004; Donaldson and Young, 2008; Veenema et al., 2013). Does a common thread run between these adaptive functions, from the standpoints of the ancestral functions of AVP, and its roles in modern human social behavior?

The purpose of this article is to propose and evaluate a simple, unified theory for the evolution and fundamental, overarching contextual function of AVP. This framework is meant to be useful for understanding not just the adaptive roles of AVP in humans, but also its effects in causing human maladaptations, which manifest in psychological and psychiatric disorders and aversive states. We first briefly describe the early, ancestral functions of AVP (and its analogs) among animals, because these, like OXT in early mammals, provide key insights into later-evolving and more diverse functions. Second, we explicate our hypothesis for the predominant role of AVP: that it specifically mediates optimal behavior during social interactions that comprise strong mixtures of cooperation with conflict. Third, we evaluate the hypothesis using data from the literature on typical human populations, as well as findings from other mammals. Fourth, we explicate the role of AVP in relation to the functions of other key hormones, namely testosterone (T), OXT, and estradiol (E2). Finally, we discuss the implications of the hypothesis for understanding autism, loneliness, and other psychological conditions.

## The Original Neuropeptides

AVP and its chemical ancestors and analogs, including vasotocin, nematocin, and others, exhibit central roles in water balance, blood pressure regulation, vasoconstriction, fear and stress reactivity, and reproduction, where they serve to guide mate searching, mate recognition and bonding, and coordinate AVP-mediated gamete or egg release with species-relevant adaptive behavior (Caldwell et al., 2008; Garrison et al., 2012; Wilczynski et al., 2017; Mainieri, 2020). Among mammals, AVP also increases sexual motivation and male erectile function (Murphy et al., 1987; Segarra et al., 1998). The roles of AVP in the coordination of physiology with sociosexual behavior are thus evident in its earliest forms, and among many diverse taxa.

Mating, and its immediate antecedents and aftermaths, almost always involve some degree of cooperation between the female and male, given the usual common goal of egg fertilization; cooperation is also common afterwards among some taxa, in the form of shared parental care (Brown, 1987). However, mating also engenders competition and conflicts between males and females (especially in situations where a female does not wish to mate, but a male does), between males (who are competing, often dangerously, to mate with the female), and sometimes between females (who compete for reproductive resources, such as territories or nests) (Arnqvist and Rowe, 2013). As such, mating is typically deeply imbued with combinations of cooperation and conflict, as well as being one of the behaviors most strongly associated with variation in fitness. Courtship and mating are thus inherently social, risky, highly rewarding, and coordinated, both physiologically and behaviorally, in notable part by AVP (Donaldson and Young, 2008; McCall and Singer, 2012).

In nematodes, insects, fishes, and many other taxa, mating is relatively simple, in terms of its cognitive control. By contrast, among mammals, and especially humans, mating typically involves an extended set of culturally embedded social behaviors, that encompass male-male competition, female choice of mates, social integration, alliance formation, social acceptance, and successful navigation of the diverse, partially divergent inclusive fitness interests of all parties involved, especially one’s mate or potential mate (Dunbar and Shultz, 2007). As such, in humans the contextualized roles of AVP in human sociosexual interactions are highly encephalized and multifaceted. Nevertheless, the essence of AVP’s function, adaptive orchestration of behavior in social and sociosexual situations that involve cooperation mixed with competition, should, by the hypothesis developed and evaluated here, remain fundamentally the same across evolutionary time.

## How and Why AVP Adaptively Coordinates Physiology With Behavior

Interacting individuals may exhibit highly coincident interests, such as a mother with an infant, highly divergent interests, such as two males competing over a mate, or interests that involve substantial components of both cooperation and competition or conflict, such as friends or relatives competing over some fitness-enhancing resource, or pair-bonded individuals in conflict over degrees of paternal investment. Our hypothesis posits that AVP is differentially involved in this latter type of interaction, where individuals are selected to negotiate balances between cooperation on one hand and competition or conflict on the other. Behaviorally, such negotiation can involve aggression, persuasion by providing benefits, coercion by imposing costs, or other mechanisms, and it can occur between any combinations of the sexes.

If the conflict-cooperation hypothesis for the predominant role of AVP in human physiology and behavior is correct, then the specific functions of AVP in human behavior, as elucidated in dozens of studies of this hormonal system, should fit within its rubric. In this section, we evaluate the degree to which this prediction is met. We focus on studies of cognition, emotion, and social behaviors, where roles for AVP have been demonstrated in humans and other mammals. In these general domains, we searched the literature in a comprehensive manner for studies that tested for roles of the AVP system in situations potentially relevant to cooperation mixed with conflict. In particular, we searched Web of Science and Google Scholar between 20 September and 31 October 2021 using combinations of the search terms “arginine vasopressin” or “vasopressin” with “cooperation,” “competition,” “cognitive,” “social,” “emotional,” or “reciprocity.” All studies that tested for effects of AVP in situations relevant to combined competition and cooperation were reported. We emphasize that this approach can demonstrate the degree to which evidence exists that is consistent with the hypothesis addressed, rather than providing a specific, systematic test, for which data are as yet unavailable. We also note that all salient studies are reported, such that fits to the main prediction are by no means preordained; for example, AVP might not be involved in specific situations like pair-bond maintenance, or social integration, where its effects are predicted.

Table 1 shows that for a diverse range of experimental and observational paradigms involving variation in the genes that codes for the AVPR1A receptor (used as a proxy for variation in AVP receptor function/sensitivity), serum, plasma or urinary AVP levels, and experimental AVP administration, this system demonstrates evidence of effects congruent with the conflict-cooperation model. In particular, AVP effects have been described for interactions that are highly social, fitness-salient, and involve both conflicts and confluences of interest. Social integration and reciprocity, and their more-basic components such as joint social attention, play central roles in these AVP functions, given that they can exhibit both positive and negative social valences on short times scales. Tests that specifically target the main predictions of this model, and compare effects of AVP with those of OXT and T in socioecologically valid situations, are required for more robust and detailed evaluation, and refinement, of this framework. For analysis of AVP and OXT in peripheral body fluids like blood and urine, standardization of assay methodologies—preferably, where appropriate for the sample fluid, a method that employs solid-phase extraction (e.g., Robinson et al., 2014)—is needed to enable robust comparisons across studies (see MacLean et al., 2019 for discussion of the challenges of measuring neuropeptides). Finally, many studies involving AVP or other neurohormones in humans are underpowered or unreplicated (e.g., Declerck et al., 2020; Quintana, 2020; Winterton et al., 2021), which cautions against overinterpretation of the results.

**TABLE 1:** Findings salient to the hypothesis that AVP mediates affect, cognition, and behavior in situations involving a mixture of cooperation and conflict.

Data source	Sample details	Main findings	Comments	References
*AVPR1A* polymorphism	1,899 adults (male/female proportions and ages not specified)	*AVPR1A* polymorphism was associated with quality of human pair-bonding and marriage, and marital status	Pair-bonding engenders highly reciprocal social interactions	Walum et al., 2008
*AVPR1A* polymorphism	151 females (age not specified)	Mothers with two copies of the RS3 allele showed less sensitive parenting, especially in environmental circumstances of high maternal early adversity	Maternal care involves some degree of parent-offspring conflict over levels of investment	Bisceglia et al., 2012
*AVPR1A* polymorphism	135 females, mean ages = 34.41 years	Mothers with the RS3 allele showed less structuring and support during interactions with their children	Maternal care involves some degree of parent-offspring conflict over levels of investment	Avinun et al., 2012
*AVPR1A* polymorphism	234 children (sex not specified), mean age 3.5 years	Children with the RS3 allele showed a reduced tendency toward altruistic behavior (in the “Dictator game”)	Interactions were among actual or potential “friends,” and so likely involve elements of reciprocity as well	Avinun et al., 2011
*AVPR1A* polymorphism	348 adults (170 males, 178 females)	RS1 alleles affected level of commitment to cooperative/altruistic civic duty, when under higher perceived threat	Clear example of cooperation and conflict	Poulin et al., 2012
*AVPR1A* polymorphism	1,871 adults (males and females), ages 20–64	Adult social integration was mediated by RS3 polymorphism and levels of childhood adversity	Social integration is probably a key factor in AVP effects	Liu et al., 2015
*AVPR1A* polymorphism	13,092 participants (males and females), ages 18–49	*AVPR1A* SNP genetic variation (gene-based test) was associated with extra-pair mating in females (though not in males)	Lack of association in males is unexpected	Zietsch et al., 2015
*AVPR1A* polymorphism (in chimpanzees)	64 captive chimpanzees (36 males, 28 females); 26 wild chimpanzees (26 males)	*AVPR1A* alleles were associated with “smart” social personality in common chimpanzees, independent of T levels	Shows continuity in AVP effects between humans and chimpanzees	Anestis et al., 2014
*AVPR1A* polymorphism	367 young adults (191 females, 176 males), mean age = 24.40	Polymorphism in *AVPR1A* was associated with cognitive empathy, not emotional empathy (using questionnaires)	Cognitive empathy involves navigation of complex social interactions	Uzefovsky et al., 2015
*AVPR1A* polymorphism	434 participants (213 males, 221 females), ages 20–59	Polymorphism alleles were associated with variation in trust and reciprocity, in a “trust game.”	Clear example of cooperation and actual or potential conflict in reciprocity. males with short allele more generous even with possibility of betrayal	Nishina et al., 2019
*AVPR1A* polymorphism (chimpanzees)	62 captive chimpanzees (43 females, 19 males)	Complexity of sociality in captive chimpanzees was associated with presence of DupB polymorphism in *AVPR1A* promoter; no relation observed for *OXTR* SNP	Shows continuity in AVP effects between humans and chimpanzees. DupB is analogous to RS3 in humans	Staes et al., 2015
*AVPR1A* polymorphism (chimpanzees)	164 captive adult and sub-adult chimpanzees (95 females, 69 males)	*AVPR1A* variants were linked to “psychopathy” (disinhibition, meanness, boldness) in captive chimpanzees	Association was not found in chimpanzees reared for first 3 years of life in “human nursery,” suggesting gene by environment interaction	Latzman et al., 2017
*AVPR1A* polymorphism (chimpanzees)	213 adult or sub-adult chimpanzees (132 females, 81 males)	Male chimpanzees with specific DupB allele in *AVPR1A* performed better on a joint attention task and were more responsive to socio-communicative cues	Polymorphism was not associated with performance in non-social task. Joint attention represents form of social integration	Hopkins et al., 2014
*AVPR1A* manipulation (mice)	Male mice	Transgenic mice with human *AVPR1a* showed increased reciprocal social interactions (sniffing and grooming of unfamiliar male partner)	Transgenic mice also showed a primate-like *AVPR1A* binding pattern; no changes in learning or memory were observed	Charles et al., 2014
Plasma AVP (measured using Assay Designs kit; extraction step is not reported)	85 (53 females, 32 males), ages 18–34 (mean age = 21.6 years)	Higher plasma AVP was associated with marital distress in males, but not in females	Lack of effect in females is unexpected	Taylor et al., 2010
Plasma AVP (measured using Enzo Life Sciences kit; extraction was not performed)	59 (31 males, 28 females), mean age = 23.8 years	Among recent migrants to a new country, increases in social integration were associated with increases in plasma AVP; baseline OXT positively predicted social relationship satisfaction, social support and reduced loneliness	Clear effect of AVP in context of social integration	Gouin et al., 2015
Plasma AVP (measured using Assay Designs kit; extraction step is not reported)	37 heterosexual married couples (37 females, 37 males), ages 22–73 years	Higher plasma AVP was associated with larger social network and fewer negative social interactions	Clear effect of AVP in context of social integration	Gouin et al., 2012
Plasma AVP (measured using Assay Designs kit; extraction step is not reported)	119 parents (71 females, mean age = 28.9 years; 48 males, mean age = 29.3 years)	Parents’ plasma AVP levels were positively associated with joint attention and stimulatory contact while interacting with their children	Joint attention represents form of social integration	Apter-Levi et al., 2014
Plasma AVP (macaques) (measured using Assay Designs kit; extraction step is not reported)	54 (29 males, 25 females) juvenile *Macaca mulatta*	Extent of social play and numbers of friendships predicted plasma AVP (but not OXT) in juvenile rhesus macaques	Social play represents competition and conflict in social-learning situation	Weinstein et al., 2014
Urinary AVP (marmosets) (solid-phase extraction followed by quantification by radioimmunoassay and HPLC)	4 adult male *Callithrix jacchus*	Urinary AVP was significantly lower in male common marmosets during social isolation; large increase in AVP when contacting another male	Social isolation removed main context for AVP system effects	Seltzer and Ziegler, 2007
AVP administration	96 males, ages 18–34	OXT but not AVP administration increased local and universal altruism	OXT but not AVP expected to mediate altruism *per se*	Israel et al., 2012
AVP administration	2012: 91 males, ages 18–22 2014: 87 females, ages 18–22	AVP administration increased reciprocation of cooperation, compared to placebo and to OXT	Clear effect of AVP in reciprocity	Rilling et al., 2012, 2014
AVP administration	Experiment 1 (Stag Hunt): 59 males, ages 19–32 Experiment 2 (Stag Hung + fMRI): 34 males, ages 19–34	AVP increased level of risky cooperative/reciprocal behavior in males (Stag Hunt game)	Clear effect of AVP in reciprocity	Neto et al., 2020
AVP administration	101 participants (53 males, 48 females), ages 18–26 (mean = 21.5)	AVP administration increased level of prosocial (providing benefits to self and ingroup members) dishonesty in females but not males	Restriction of effect to females may involve increased female within-group bonding	Feng et al., 2020
AVP administration	152 males, ages 18–35 (mean age = 25.1 years)	AVP increased levels of social stress in a socially evaluative situation (Trier Social Stress test)	AVP mediates social challenge	Shalev et al., 2011
AVP administration	48 males, age 18–60 (mean 22)	AVP enhanced memory of happy and angry faces in male participants	By altering salience of social cues, AVP may facilitate both bonding and aggression	Guastella et al., 2010
AVP administration	38 males, 38 females, ages 17–25	In males, AVP administration promoted agonistic responses to images of unfamiliar males; in females, AVP promoted affiliative responses to images of unfamiliar females	AVP may mediate different balances of negative and positive responses to stressful social scenarios in males compared to females	Thompson et al., 2006
AVP administration	48 males, 51 females, ages 18–26	In males, AVP decreased friendly responses to images of neutral/positive male faces; in females, AVP increased friendly responses to negative female faces	AVP may mediate different balances of negative and positive responses to stressful social scenarios in males compared to females	Wu et al., 2019
AVP administration	304 adults (153 males; 151 females), ages 18–22	In Prisoner’s Dilemma Game, AVP administration increased reciprocity (tit-for- tat) over all-cooperation; AVP also increased sensitivity to behavior of others	Clear effect of AVP in reciprocity	Neto et al., 2020

*To be included, studies must use a paradigm that explicitly or implicitly involves cooperation and conflict, or that allows differentiation of the effects of the two for AVP and OXT in this context.*

*Studies include analyses using AVP intranasal administration; measurement of AVP in plasma or urine; and analysis of genetic polymorphisms in the AVPR1A receptor gene. For studies quantifying AVP in plasma or urine, methodology is also reported with regard to whether an extraction step was performed.*

*AVP, arginine vasopressin; AVPR1a, arginine vasopressin 1a receptor gene; HPLC, high-performance liquid chromatography; OXT, oxytocin; OXTR, oxytocin receptor gene; SNP, single nucleotide polymorphism.*

## The Combined Roles of AVP, OXT, T, and E2

**FIGURE 1:**
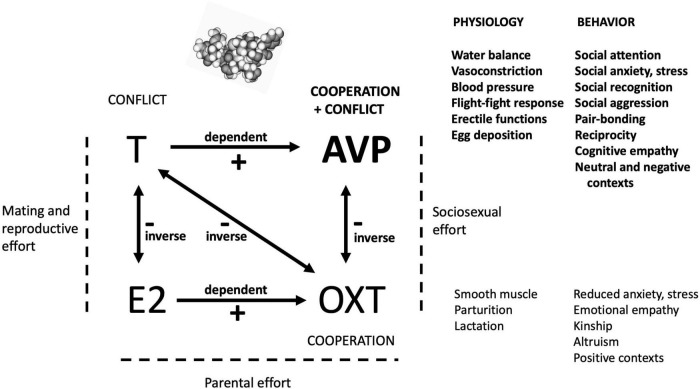
A highly simplified diagram of the postulated primary effects of the four hormones analyzed here. By the hypothesis presented and evaluated in this study, AVP has evolved, from its simple ancestral physiological and behavioral functions, to orchestrate complex social cognition and behavior that involves mixtures of cooperation with conflict. The mediation of social and sociosexual situations by AVP depends upon levels of other hormones, as shown here. AVP is ideally placed in this broader context, given its dependence on T, its inverse relationship with OXT, and its effects as a neurotransmitter as well as a hormone. The roles of E2 in social-behavioral neuroendocrinology are insufficiently characterized for robust inferences regarding its specific functions in this domain to be drawn.

How does the primary function of AVP described here relate to the functions of other hormones central to human social and sexual behavior? Affect, cognition and behavior are modulated, after all, not by levels and reactivities of single hormones, but through sets of neuropeptide and steroid hormones acting together (Van Anders et al., 2011; Gabor et al., 2012; Neumann and Landgraf, 2012; Schneider et al., 2021); for example, under an emotional empathy-inducing stimulus, salivary OXT levels go up while T levels decline (Procyshyn et al., 2020). Figure 1 summarizes the postulated predominant roles of AVP, OXT, E2 and T in relation to one another, and, for AVP and OXT, in relation to their physiological antecedents and underpinnings.

The functions of these four hormones can usefully be subsumed into the main forms of effort expended during an individual’s life history (Alexander, 1979). First, T and E2 mediate mating and reproductive effort via coordination of sexual behavior with fitness-relevant physiology, for the gonads, secondary sexual traits, and brain, with male and female biases for the two hormones, respectively. T subserves competition and conflict, usually over mates, resources or status; the genetic basis of its serum levels is independent in the two sexes as indicated by recent GWAS data (Sinnott-Armstrong et al., 2021), and inverse effects with E2 are subserved by aromatase. T may also mediate benevolence, but it is only predicted to do so in situations in which such behavior increases the status, and thus the perceived competitive abilities, of the actor (e.g., Dreher et al., 2016).

Second, AVP and OXT mediate social effort, defined here as behavior focused on the acquisition, navigation and use of social supports and alliances involving non-kin (friends or allies) and/or kin. The social effects of AVP and OXT are neurological, with an inverse relationship generated by the conflict (and associated anxiogenic) dimension in AVP effects, in contrast to the prominent cooperation and anxiolytic effects of OXT (Motoki et al., 2016; Plasencia et al., 2019). Third, parental effect in child-rearing is mediated mainly by E2 and OXT. OXT effects are indeed dependent on E2 (e.g., Acevedo-Rodriguez et al., 2015), whereas AVP effects are enhanced by T (Delville et al., 1996; Auger et al., 2011) and thus exert male-biased impacts (Goodson and Bass, 2001; Dumais and Veenema, 2016). In this context, maternal care is mainly controlled by OXT, but AVP may also be involved given maternal-offspring conflicts over optimal levels of maternal investment (Trivers, 1974).

Friendship, bonding, and their antithesis in loneliness, are inherently social, and so should be underlain mainly by effects of AVP (especially for male-to-female bonding, and male to male bonding) and OXT (especially for female to female bonding, and female to male bonding). By contrast, bonding of either parent to offspring appears mediated mainly, though not exclusively, by OXT. Male to female bonding in humans engenders cooperation in reproduction and child-rearing, but the cooperation is fraught with conflicts due to three factors: (1) selection for extra-pair mating especially in males, (2) selection for male control of female reproduction, and (3) selection for females to constrain male promiscuity, and control their own reproductive options and the resources that they require to maximize fitness. AVP has indeed been well-demonstrated to underpin variation in male to female pair bonding, and promiscuity-parental tradeoffs, in humans and non-human mammals (e.g., Okhovat et al., 2015).

Male to male bonding is rare among mammals, being found, among non-humans, mainly in complexly social species, including lions, bottlenose dolphins, and common chimpanzees, where groups of males cooperate for dominance over other male coalitions and for control of females and their reproduction (Whitehead and Connor, 2005). Such alliances can be long-lasting and highly cooperative and reciprocal, but are also imbued with competition for status and mating opportunities. As such, male-male alliance, bonding, and “friendship” are expected to be driven mainly by AVP, and by OXT as well only to the extent that the success of male coalitions, in competition with other coalitions, depends strongly on the closeness of male interdependency and mutual support (Flinn et al., 2012). There is a notable lack of studies on the hormonal basis of male-male bonding in humans and other mammals; however, for juvenile rhesus macaque males, numbers of proximity-based friendships, play-based friendships, reciprocal friendships, and overall number of friendships are positively associated with levels of plasma AVP (Weinstein et al., 2014). AVP also mediates social play in juvenile rodents (Veenema et al., 2013; Paul et al., 2014), with such play representing a clear manifestation of conflict mixed with cooperation especially among males.

The separate and combined roles of AVP, OXT, E2 and T are not independent of sex, given the differences between males and females in the nature of their optimal, evolved social and sexual interactions. AVP thus shows divergent effects in male and females for some phenotypes, being associated, for example, with increased agonistic facial motor patterns in human males but increased affiliative patterns in females, after intranasal administration (Thompson et al., 2006). Such differences can be attributed to between-sex variation in optimal sociosexual strategies, with female behavior tending to involve more affiliation and cooperation than competition (especially under threat), while male behavior is under selection for higher levels of antagonism and less cooperation (Taylor, 2006). By the conflict-cooperation model, sex differences in AVP effects should be more quantitative and context-dependent than qualitative, with females subject to cooperation-conflict situations tilted more toward cooperation. That said, few studies have yet been conducted of AVP effects in females under ecologically realistic conditions with competition and cooperation both present.

Finally, the diverse functions of AVP are associated with distinct AVP receptor types (AVPR1a, AVPR1b, and AVPR2), with only the later two linked with social behavior (Baribeau and Anagnostou, 2015). Moreover, AVP can also bind to OXT receptors, and vice versa (Song and Albers, 2018). Despite awareness of such cross-talk, it is rarely considered in experimental designs. Given that OXT and AVP are thought to have complex, overlapping, and sometimes opposing effects, this cross-talk poses a challenge for the ability of researchers to tease apart the effects of specific neuropeptides in specific aspects of emotion, behavior or psychological conditions. Such crosstalk is, however, consistent with the hypothesis described here, in that AVP and OXT overlap in some of their effects on social cooperation, and at least in the case of maternal defensiveness, OXT can also be involved in antagonistic behavior (e.g., Bosch, 2013).

## Loneliness, Autism, AVP, and OXT

If AVP is fundamental for successful navigation of social challenges and opportunities, then individuals with a more contextually, adaptive reactive and responsive AVP system are predicted to be more socially successful. By contrast, dysfunction of the AVP system is predicted to manifest as the inability to handle complex social situations and successfully integrate into groups, resulting in emotions like loneliness or psychological conditions such as autism that are characterized by social problems.

Autism was originally described by Kanner (1943) as a disorder of affective (social-emotional) contact. It is highly heterogeneous, with considerable variation in its genetic causes (from *de novo* mutations of large effect, to polygenic inheritance), and its spectrum of phenotypic effects (e.g., from comorbid intellectual disability to high intelligence). Loneliness is expected to exhibit partial overlap with autism (in relatively high-functioning individuals), in that it results from a lack of affective contact combined with unfulfilled social motivation, which may or may not be present in any given individual with autism. Loneliness also differs from autism in that it represents an aversive state that can have adaptive effects (Nesse, 2019), to the degree that it compels individuals to change their behavior and seek out pleasurable and satisfying affiliations.

How do these conceptualizations of loneliness, and autism, relate to theory and evidence for the contextual adaptive significance of AVP and OXT? OXT is expected to predominantly involve cooperation and positive social bonding, in relatively simple social situations. As such, activity of this hormone is expected to directly and effectively ameliorate the painful aspects of loneliness. By contrast, a well-functioning AVP system, given its central role in more-complex social situations involving mixtures of cooperation and competition or conflict, is involved in social navigation and integration (Gouin et al., 2015; Liu et al., 2015; Table 1), which are required for long-term social success. As such, a well-functioning AVP system is expected to alleviate loneliness indirectly but in an enduring manner.

These inferences fit with the data showing: (1) that AVP levels are significantly lower in cerebrospinal fluid of children with autism (Oztan et al., 2018, 2020), and in primate models of autism (Parker et al., 2018); (2) that AVP administration can alleviate social symptoms of autism in mice and rats (Borie et al., 2021; Wu et al., 2021) and in children (Hendaus et al., 2019; Parker et al., 2019), (3) that the AVP system is specifically involved in social joint attention (Apter-Levi et al., 2014; Hopkins et al., 2014) and social recognition (Bielsky and Young, 2004), that are selectively reduced in individuals with autism (Bruinsma et al., 2004); and (4) that social isolation strongly modulates AVP system effects, in rodents (e.g., Ross et al., 2019). Despite these commonalities, some studies do not fit clearly with such a simple paradigm; for example, blocking the AVPR1A receptor was associated with higher socialization and communication scores in one study of autistic adult men (Bolognani et al., 2019). The precise neuroendocrine effects of AVP and its receptors in autism etiology and traits thus remain unclear.

As regards loneliness, a key question then becomes whether it represents: (1) a temporary state brought on by external social circumstances, (2) a longer-lasting trait mediated by endocrine systems and their underlying neurogenetic bases, or (3) some combination or interaction of state with trait. Temporary, state-based loneliness represents, as noted above, a conditionally adaptive aversive condition, in that individuals lacking in social support or friends who did not feel lonely (and so did not seek to alleviate it) are expected to suffer fitness-relevant costs. By contrast, trait-based loneliness is much more closely akin to autism, and should, by the hypothesis and evidence described here, involve alterations to the AVP and/or OXT systems that are maladaptive and present from birth or environmentally induced. The degree to which such longstanding challenges can be ameliorated pharmacologically, such as via neuropeptide receptor modification or via psychological therapies, remains to be seen.

## Conclusion

The dictum “*natura non facit saltus*” refers to the continuity in structure and function required by evolution under natural selection. In this article, we have argued that the ancestral functions of AVP provide key insights into its current main role in modulation of human sociality: the navigation of social and sociosexual situations that combine the opportunities of cooperation with the threats of conflict and competition.

The main upshots of these considerations are threefold. First, our hypothesis for the adaptive significance of AVP in humans requires ecologically valid tests, to determine if endogenous AVP levels fluctuate as predicted in response to relevant circumstances. These and other tests of the cooperation-conflict hypothesis for AVP should also consider trajectories in levels of other relevant hormones (especially OXT, E2, T, and cortisol as an index of stress) operating jointly with inverse or positively coordinated effects (Figure 1). Second, the AVP system is predicted to be associated with loneliness, through its effects on the ability of individuals to successfully manage complex social situations and thereby integrate successfully in a social dyad or group, be it with friends, mates, offspring, other relatives, colleagues or acquaintances. This integration represents Bowlby’s attachment system writ large during adulthood (Carter, 2017), and focuses on avenues of striving to increase one’s inclusive fitness. Third, the roles of the AVP system in neurodevelopmental disorders, especially autism and schizophrenia, are only beginning to be explored. Recent replicated studies showing low cerebrospinal AVP among individuals with autism indeed suggest a key role for this hormone in autism-related phenotypes (Oztan et al., 2018, 2020; Parker et al., 2018), and longstanding reports of high AVP in acute schizophrenia or psychosis (Raskind et al., 1978; Goldman et al., 1997; Rubin et al., 2013; Guzel et al., 2018) suggest unrecognized impacts in this disorder as well.

Most importantly, studies of AVP in humans require a robust theoretical framework that explains the adaptive significance of the system, and that thereby allows for predictive tests, and inferences concerning what adaptive phenotypes exhibit maladaptive expression in any given disorder or conditions, for any given individual. Like nature, human social development does not make jumps—and its progress is guided by the steroid and peptide hormones that collectively steer our cognition, affect, and behavior.

## Data Availability Statement

The original contributions presented in the study are included in the article/supplementary material, further inquiries can be directed to the corresponding author.

## Author Contributions

BC wrote the manuscript. BC, TP, and MM edited the manuscript. All authors conceived the manuscript and conceptualized the model.

## Conflict of Interest

The authors declare that the research was conducted in the absence of any commercial or financial relationships that could be construed as a potential conflict of interest.

## Publisher’s Note

All claims expressed in this article are solely those of the authors and do not necessarily represent those of their affiliated organizations, or those of the publisher, the editors and the reviewers. Any product that may be evaluated in this article, or claim that may be made by its manufacturer, is not guaranteed or endorsed by the publisher.
